# The pterygomaxillary junction: An imaging study for surgical information of LeFort I osteotomy

**DOI:** 10.1038/s41598-017-10592-8

**Published:** 2017-08-30

**Authors:** Yen-Po Chin, Maria Belen Leno, Sarayuth Dumrongwongsiri, Kyung Hoon Chung, Hsiu-Hsia Lin, Lun-Jou Lo

**Affiliations:** 1 Department of Medicine, National Yang Ming University, Taipei, Taiwan; 2 Plastic & Reconstructive Surgery, and Craniofacial Research Center, Chang Gung Memorial Hospital, Chang Gung University, Taoyuan, Taiwan; 3 Craniofacial Research Center, Chang Gung Memorial Hospital, Taoyuan, Taiwan

## Abstract

Maxillary osteotomy is a common surgical procedure and often involves separation of the pterygomaxillary junction (PMJ), which is a “blinded” procedure with inherent risks. Knowledge of the PMJ structure is essential. It remains unclear whether patients with different facial types have different PMJ structures, or different surgical outcome. This study evaluated the computed tomographic images of 283 consecutive patients who received orthognathic surgery. Patients were classified into Angle class I, II, III and cleft lip/palate groups. The results showed that the PMJ was 5.1 ± 1.4 mm in thickness, 9.7 ± 1.7 mm in width, and 102.0 ± 4.0 degrees relative to the sagittal plane in the level of posterior nasal spine. There were no statistically significant differences in these measurements among the groups. The class III group demonstrated significantly smaller angle relative to the maxillary occlusal plane. The cleft group showed significantly longer vertical distance between the posterior nasal spine and the lower border of PMJ, shorter distance between the second molar and PMJ, and longer distance between the descending palatine artery and PMJ. With regard to postoperative outcome, the cleft group showed higher incidence of pterygoid plate fracture. The results in this study provide additional surgical anatomic information.

## Introduction

Maxillary LeFort I osteotomy is a widely applied procedure in the surgical management of dentofacial deformities, trauma, as well as tumors in the skull base and midfacial regions^1, 2^. Nevertheless, separation of the pterygomaxillary junction (PMJ) during the osteotomy is a challenging procedure for most surgeons, as it sits behind the maxilla and is not directly visible. While most of the LeFort I related complications could be avoided by an adequate planning and implementation, the PMJ separation is a blind maneuver and technically risky, especially in patients with craniofacial deformities.

During the pterygomaxillary osteotomy, the pterygoid plate should remain intact^3^. A careless attempt to separate the PMJ could result in untoward pterygoid plate fracture^4–7^, vascular or neural complications^8–14^, and even blindness^15–17^. The patterns of pterygoid plate fractures have been evaluated in radiographic, cadaveric, and dry skull studies^6, 18–22^. However, these studies were mostly made in normal samples, instead of patients with malocclusion or with cleft lip and palate deformities. Likewise, fracture occurring in front of the PMJ causes surgical difficulty in maxillary movement. Patients with cleft deformity have been proven to be at relatively high risk for the complications related to atypical anatomy^23^. Due to the high prevalence of requiring LeFort I osteotomy in patients with cleft lip and palate deformity, malocclusion, or facial asymmetry^24–26^, accurate surgical disjunction of the PMJ can prevent unexpected complications.

Several methods have been described in the literature to maximize patient safety and prevention of complications since Turvey first described recommendations to safely perform the LeFort I osteotomy^27^. In the literature, operative techniques varied with using an oscillating saw, an endoscope approach, or avoiding direct PMJ separation by using various techniques^28–35^. Some techniques were markedly time-consuming, surgeon dependent, or relied on the availability of specific instruments. However, the results have not been consistent regardless of the approach^36, 37^.

A thorough understanding of the PMJ anatomy is mandatory in patients requiring LeFort I osteotomy. The literature remains unclear whether different types of craniomaxillofacial deformity affect the PMJ structure or intra-operative pterygoid plate fracture. In this study, cone-beam computed tomogram (CBCT) images of 283 consecutive patients undergoing orthognathic surgery were analyzed. This information should provide surgeons with a better understanding of the PMJ region.

## Results

**Figure 1:**
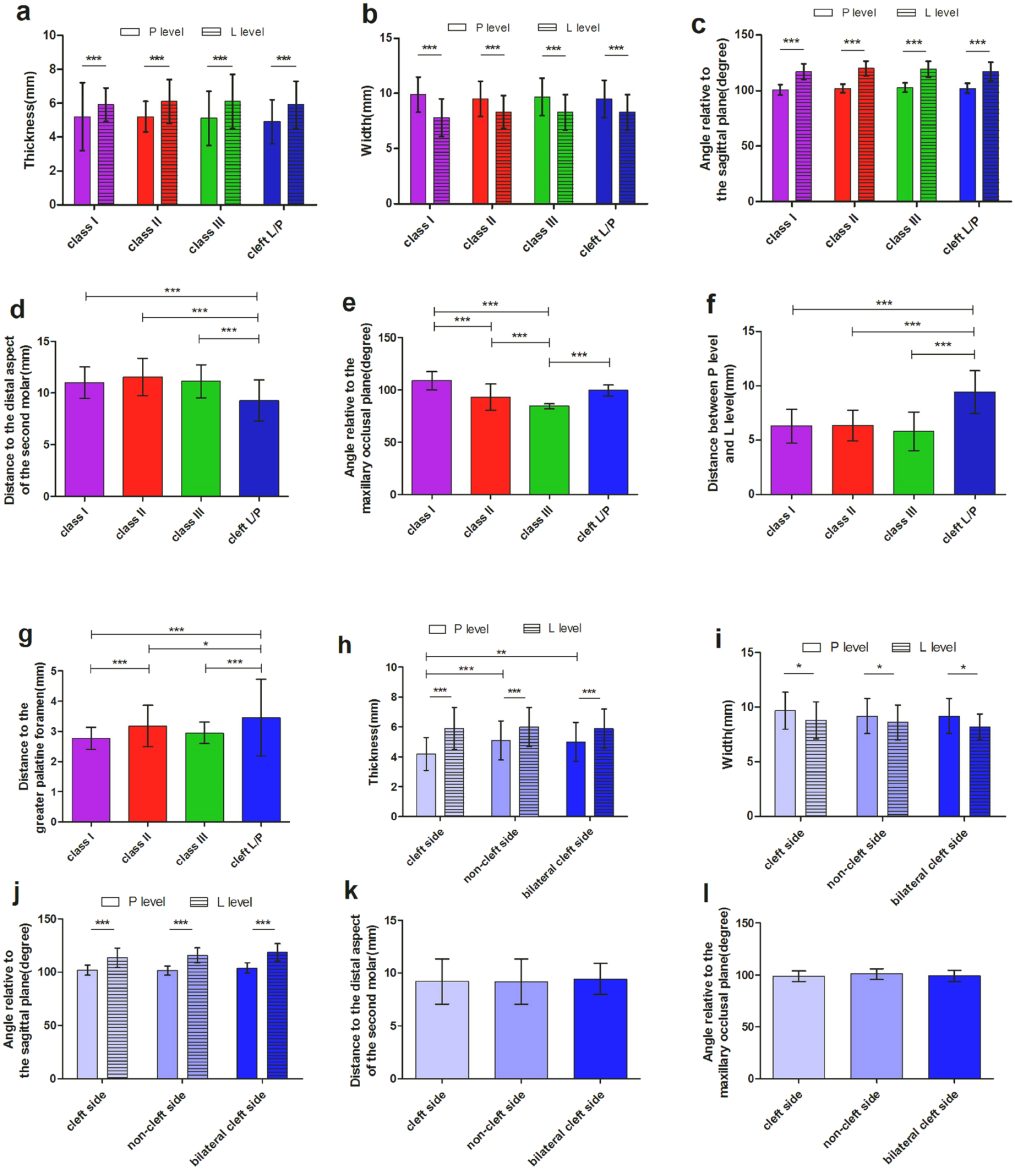
The comparisons of different anatomic variables of pterygomaxillary junction. (**a**) Comparison of the thickness between the posterior nasal spine level (P level) and the lower border of the pterygomaxillary junction level (L level), and between the Angle class I, II, III and cleft groups. (**b**) Comparison of the width between P level and L level, and between the four groups. (**c**) Comparison of the angle relative to the sagittal plane between P level and L level, and between the four groups. (**d**) Comparison of the distance between the pterygomaxillary junction and the second molar between the four groups. (**e**) Comparison of the angle relative to the maxillary occlusal plane between the four groups. (**f**) Comparison of the distance between P level and L level between the four groups. (**g**) Comparison of the distance between greater palatine foramen and pterygomaxillary junction between the four groups. (**h**) Comparison of the thickness between P level and L level, and between cleft side, non-cleft side and bilateral cleft side. (**i**) Comparison of the width between P level and L level, and between cleft side, non-cleft side and bilateral cleft side. (**j**) Comparison of the angle relative to sagittal plane between P level and L level, and between cleft side, non-cleft side and bilateral cleft side. (**k**) Comparison of the distance between the pterygomaxillary junction and the distal aspect of the second molar between cleft side, non-cleft side and bilateral cleft side. (**l**) Comparison of the angle relative to the maxillary occlusal plane between cleft side, non-cleft side and bilateral cleft side. Statistical analysis was performed by one-way or two-way ANOVA test. Error bars represent standard deviation. *p < 0.05; **p < 0.01; *** p < 0.001.

The patient numbers, age, and gender of these groups were listed as following: 62 patients (17 M 45 F, mean age 26.1 ± 5.1 years, range 15 to 38 years) in the Angle classification class I group, 68 patients (12 M 56 F, mean age 26.0 ± 6.2 years, range 15 to 39 years) in the class II group, 84 patients (42 M 42 F, mean age 23.3 ± 5.0 years, range 17 to 38 years) in the class III group, and 69 patients (39 M 30 F,mean age 18.0 ± 2.4 years, range 15 to 32 years) in the cleft lip/palate group. Demographic and anatomic variables for each group were summarized in Table 1. For further analysis in the cleft group, we divided the cleft group into the cleft side group, the non-cleft side group, and the bilateral cleft side group. The demographic and anatomic variables were demonstrated in Table 2. In general, the PMJ was 5.1 ± 1.4 mm in thickness, 9.7 ± 1.7 mm in width, and 102.0 ± 4.0 degrees relative to the sagittal plane in the level of posterior nasal spine. There were no statistically significant differences in these measurements (*p* > 0.05, ANOVA test) among the Angle class I, class II, class III and the cleft group at the posterior nasal spine level (P level) and the lower border of the PMJ level (L level) (Table 1). Statistically significant differences in the thickness, width and the angle relative to the sagittal plane were noted between the P level and L level (all *p* < 0.001, ANOVA test) (Fig. 1a to c).There was statistically significant difference in the class III group in the angle relative to the maxillary occlusal plane comparing with other three groups (*p* < 0.001, ANOVA test) (Fig. 1e).The distance to the distal aspect of the second molar of the cleft group was significantly shorter (*p* < 0.001, ANOVA test) (Fig. 1d), the distance to the greater palatine foramen of the cleft group was significantly longer (*p* < 0.001, ANOVA test) (Fig. 1g), and the distance between the P level and the L level of the cleft group was significantly longer (*p* < 0.001, ANOVA test) compared to the other three groups (Fig. 1f).

**Table 1:** Demographic and anatomic variables of the pterygomaxillary junction of Angle class I, class II, class III and cleft patients.

	Class I	Class II	Class III	Cleft	Total	p value AVONA
Demographic variables						
Sample (sides)	124	136	168	138	566	
Male/Female	17/45	12/56	42/42	39/30	110/173	
Mean age (year)	26.1 ± 5.1	26.0 ± 6.2	23.3 ± 5.0	18.0 ± 2.4	23.5 ± 4.8	
Anatomic variables						
Posterior nasal spine level						
Thickness (mm)	5.2 ± 2.0	5.2 ± 0.9	5.1 ± 1.6	4.9 ± 1.3	5.1 ± 1.4	p > 0.05
Width (mm)	9.9 ± 1.6	9.5 ± 1.6	9.7 ± 1.7	9.5 ± 1.7	9.7 ± 1.7	p > 0.05
Angle relative to the sagittal plane (degree)	100.7 ± 4.8	104.0 ± 4.0	102.1 ± 4.3	102.0 ± 4.6	102.0 ± 4.0	p > 0.05
Lower border of the pterygomaxillary junction level						
Thickness (mm)	5.9 ± 1.0	6.1 ± 1.3	6.1 ± 1.6	5.9 ± 1.4	6.0 ± 1.4	p > 0.05
Width (mm)	7.8 ± 1.7	8.3 ± 1.5	8.3 ± 1.6	8.3 ± 1.6	8.2 ± 1.6	p > 0.05
Angle relative to the sagittal plane (degree)	117.1 ± 6.9	120.0 ± 6.7	119.3 ± 7.1	115.1 ± 8.6	118.1 ± 7.3	p > 0.05
Other measurements						
Distance between P level and L level (mm)	6.3 ± 1.5	6.3 ± 1.4	5.8 ± 1.8	8.4 ± 2.0	6.4 ± 1.9	p < 0.001
Distance to the distal aspect of the second molar (mm)	11.0 ± 1.5	11.6 ± 1.8	11.1 ± 1.6	9.3 ± 2.0	10.9 ± 1.8	p < 0.001
Distance to greater palatine foramen (mm)	2.8 ± 1.9	3.2 ± 0.6	3.0 ± 0.3	3.4 ± 1.0	3.1 ± 0.8	p < 0.001
Angle relative to the maxillary occlusal plane (degrees)	108.7 ± 6.4	93.2 ± 9.2	84.4 ± 2.0	99.6 ± 5.1	95.0 ± 5.5	p < 0.001

All data showed in mean ± SD. P level = posterior nasal spine level. L level = lower border of the pterygomaxillary junction level.

**Table 2:** Demographic and anatomic variables of the pterygomaxillary junction of cleft side and non- cleft side from unilateral cleft patients, and also bilateral cleft sides from bilateral cleft patients.

	Cleft side	Non-cleft side	Bilateral cleft side	p value ANOVA
Demographic variable				
Sample(sides)	52	52	34	
Male/Female	32/20	32/20	6/11	
Mean age (year)	18.0 ± 2.4	18.0 ± 2.4	18.0 ± 3.4	
Anatomic variable				
Posterior nasal spine level				
Thickness (mm)	4.2 ± 1.1	5.1 ± 1.3	5.0 ± 1.3	p < 0.001
Width (mm)	9.7 ± 1.7	9.7 ± 1.6	9.2 ± 1.6	p > 0.05
Angle relative to the sagittal plane (degree)	101.7 ± 4.7	101.4 ± 4.4	103.8 ± 4.8	p > 0.05
Lower border of the pterygomaxillary junction level				
Thickness (mm)	5.9 ± 1.4	6.0 ± 1.3	5.9 ± 1.3	p > 0.05
Width (mm)	8.8 ± 1.7	8.6 ± 1.6	8.2 ± 1.2	p > 0.05
Angle relative to the sagittal plane (degree)	113.4 ± 9.0	115.7 ± 7.0	118.6 ± 8.2	p > 0.05
Other measurement				
Distance to the distal aspect of the second molar (mm)	9.2 ± 2.1	9.2 ± 2.1	9.5 ± 1.5	p > 0.05
Angle relative to the maxillary occlusal plane (degree)	98.8 ± 5.1	100.8 ± 5.2	99.0 ± 5.5	p > 0.05

All data showed in mean ± SD. P level = posterior nasal spine level. L level = lower border of the pterygomaxillary junction level.

For the analysis within the cleft group, the thickness of the cleft side group at the P level was significantly thinner compared to that of the non-cleft side group and the bilateral cleft side group (*p* < 0.001 and *p* < 0.01 each, ANOVA test) (Fig. 1h). Significant differences were found in the thickness, width and angle relative to the sagittal plane between the P level and the L level (*p* < 0.001, *p* < 0.05 and *p* < 0.001 respectively, ANOVA test) (Fig. 1h, i and j). There was no significant difference found in the width and angle within cleft groups (*p* > 0.05, ANOVA test) (Fig. 1i). There were also no significant differences found in the distance to the distal aspect of the second molar and the angle relative to the maxillary occlusal plane in the analysis within cleft group (*p* > 0.05, ANOVA test) (Fig. 1j and k).

The postoperative CBCT axial images were used at the P level to study the pterygomaxillary separation type after LeFort I osteotomy. Postoperative images of the Angle class I group (24 images, 48 sides), class II group (53 images, 106 sides), class III group (84 images, 168 sides) and the cleft lip/palate group (69 images, 138 sides) were collected (Table 3). The cleft lip/palate group was composed of unilateral cleft lip/palate group (52 images, 52 cleft sides, 52 non-cleft sides) and bilateral cleft lip/palate group (17 images, 34 bilateral cleft sides) (Table 4). The fractures were classified into three types including clean-cut type, maxillary sinus type, and pterygoid plate type. The clean-cut type involved the most portions in the class I, class II, class III and the cleft group (60%, 69%, 67% and 71%, respectively). For the unfavorable fracture types, among these four groups, the percentage of the maxillary sinus type was the highest in the Class III group (22%), and the percentage of the pterygoid plate fracture type was the highest in the cleft group (23%). Different groups were found to have significant association with the separation types (Chi-square test, p = 0.0013). As for the analysis within the cleft group, the percentage of the pterygoid plate fracture type was the highest in the cleft side group (36%). Again, different cleft groups were significantly associated with the separation types (Fisher exact test, p = 0.0387). No severe complications such as hemorrhage or cranial base fracture were noted in the images or patient’s medical records.

**Table 3:** Pterygomaxillary junction separation types in postoperative CT at the posterior nasal spine level of Angle’s classification I, II, III and cleft patients.

	Class I	Class II	Class III	Cleft
Total sides	48	106	168	138
Clean-cut type	29 (60%)	73 (69%)	113 (67%)	98 (71%)
Maxillary sinus type	9 (19%)	17 (16%)	37 (22%)	8 (6%)
Pterygoid plate fracture type	10 (21%)	16 (15%)	18 (11%)	32 (23%)

(Chi-square test, p = 0.0013).

**Table 4:** Pterygomaxillary junction separation types in postoperative CT at the posterior nasal spine level of cleft side and non-cleft side from unilateral cleft patients, and also bilateral cleft sides from bilateral cleft patient. (Fisher exact test, p = 0.0387).

	Cleft side	Non-cleft side	Bilateral cleft
Total sides	52	52	34
Clean cut type	29 (56%)	42 (81%)	27 (79%)
Maxillary sinus type	4 (8%)	3 (6%)	1 (3%)
Pterygoid plate fracture type	19 (36%)	7 (13%)	6 (18%)

## Discussion

Separation of pterygomaxillary junction is a critical step during LeFort I osteotomy to enable complete movement of the maxilla. However, this step carries risks due to its blind surgical approach. An unfavorable pterygomaxillary separation may cause restricted mobility of the maxillary segment, pterygoid plate fracture, vascular or neural complications, or blindness. In an attempt to avoid these complications, some authors perform the osteotomy through the maxillary tuberosity, as described by Trimble^35^. They asserted that it reduced unfavorable fractures of the pterygoid plates^36^ compared to traditional disjunction, and also increases the safety margin to the structures of the sphenopalatine fossa, reducing the incidence of hemorrhages^21, 35^. One drawback is that the method can only be performed if the wisdom teeth are removed. The other one is that the course of the osteotomy, by moving apart from the pterygopalatine fossa, may come nearer to the descending palatine artery, which is a vulnerable source of bleeding during the LeFort I osteotomy^3^. Ligation of the descending palatine artery does not necessarily jeopardize the blood flow to the maxillary segment, as the gingival blood flow could remain the same with or without preservation of the artery^38^. It is advised to carefully perform the osteotomy to prevent injuring the vessel in order to avoid excessive bleeding and other unfavorable complications^39^. To achieve this goal, an identification method for the descending palatine artery during the osteotomy would be necessary.

Full knowledge of the anatomic structures in this region is essential for surgeons performing the procedure. While the posterior maxilla has been previously studied in normal human skull samples, the specific features in patients with different malocclusions, if any, were yet to be characterized. This fact seems to be important for two reasons. First, there is at least one type of abnormal maxillary morphology that has proved to be at high risk of neurovascular complications^16^. Second, there seems to be a high inconsistency in results among individuals from different studies when the same method was used for disjunction, raising doubts whether the anatomy of the patient itself is a factor. It is worthwhile noting that previous studies regarding the ideal positioning of the osteotomy only mentioned its angulation in one of the planes of the space, randomly selecting or even neglecting other planes which may have different anatomic variables. Therefore, results and conclusions can be inadvertently biased. This is likely the cause of such diverse or even contradictory results in previous studies and makes them less suitable for comparison.

The main challenge for surgeons during the PMJ separation is finding, without direct vision, the location of the pterygomaxillary junction from its lateral side. It is typically done by detecting a concavity in the pterygomaxillary fissure with a curve osteotome. Some patients may have less obvious or undefined PMJ. In this case, it could be helpful to have an idea of the location by knowing the distance from the most distal aspect of the second molar. We selected this structure as a landmark and guide because it is the most posterior identifiable hard structure in the surgical field, and its location is practically constant at an average of 10.9 mm, ranging from 9.3 to 11.6 mm. Our study showed a significantly shorter distance in the cleft lip/palate groups, averaging 9.3 mm. In the rest of the groups, we did not find statistically significant differences, averaging 11.0, 11.6, and 11.1 mm for the class I, class II and class III groups respectively. This represents the distance in a straight line parallel to the sagittal plane and easy to estimate by the surgeon in the operating room.

**Figure 2:**
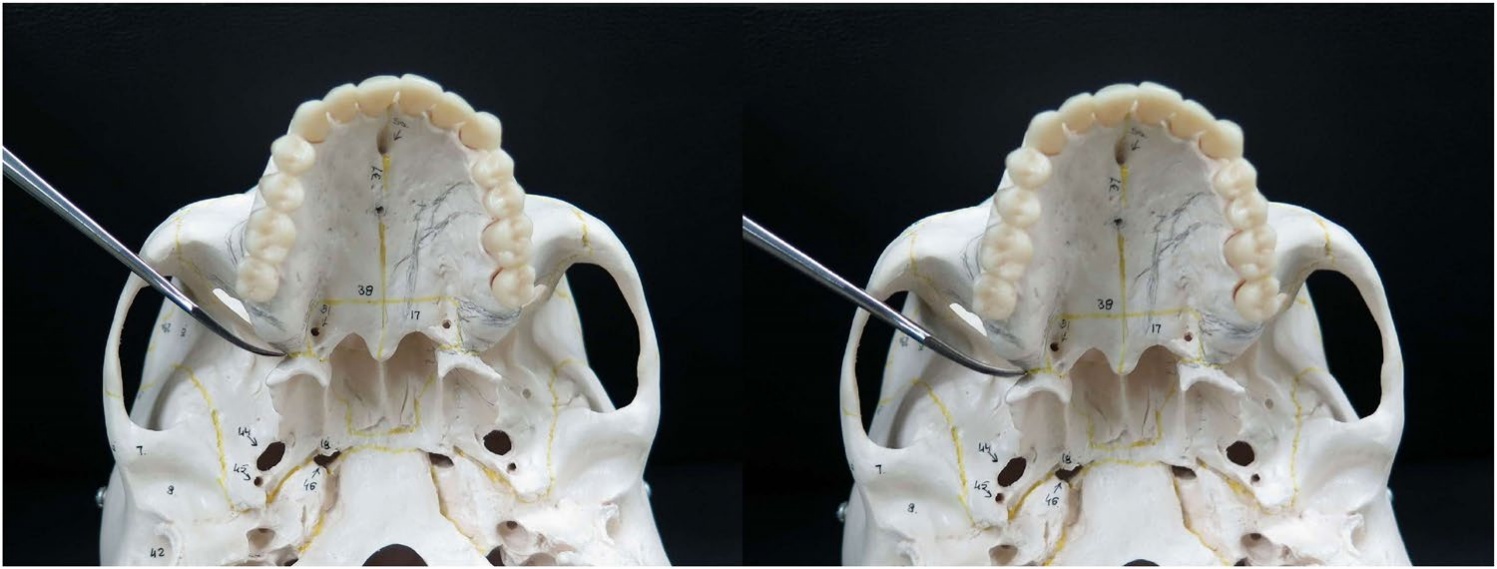
Placement of curved osteotome in the pterygomaxillary junction. Left: 90 degrees to the sagittal plane. Right: 102 degrees to the sagittal plane, a preferred angulation for smooth separation of the pterygomaxillary junction.

The ideal separation line begins laterally in the pterygomaxillary groove, and progresses medially along the pterygomaxillary junction between the maxilla and the lateral pterygoid process. The lateral to medial course of the osteotomy in this region is fairly unpredictable and this line creates an angle with the sagittal plane that is greater than 90° (not perpendicular). One of the previous studies measured a similar angle, concluding it was around 104° in average in Thai skulls^6^. Knowing this angulation beforehand guides the surgeon when placing the osteotome, and therefore avoids osteotomies progressing too anteriorly and approaching the descending palatine artery, or too posteriorly fracturing the pterygoid plate^16^. Previous recommendations have been made of using the osteotome or saw in approximately 90° relative to the sagittal plane^28, 30, 40^. Our study found that the angle created by the junction relative to the sagittal plane is, by mean, 100.7°, 104.0°, 102.1° and 102.0° at the posterior nasal spine level, and 117.1°, 120.0°, 119.3°, 115.1° at the lower border of the PMJ level in patients with the Angle class I, II, III and cleft lip/palate group respectively. There was no statistically significant difference between non-cleft and cleft patients. Therefore, it is reasonable to believe that the tip of the instrument used for the separation should be placed with this angulation, which is greater than the previous recommendation. A limitation of our recommendation is that it may be difficult to evaluate the angulation of the instrument in the operating room, especially with curved osteotome. However, a rough estimation and practice of the osteotomy direction can be made (Fig. 2).

Our results are in accordance with those from Stajcic^41^. He proved that by increasing the osteotome angulation from the conventional 50° to 80°, the incidence of unwarranted fractures of the pterygoid plates decreased significantly. However, the fractures of both groups were all low-level fractures, and therefore its clinical significance is questionable.

In the sagittal plane, the distance between the posterior nasal spine and the lower border of the pterygomaxillary junction were measured. It allowed an idea of how inferior the end of the junction is located, once the surgeon identifies the posterior nasal spine. It averaged 6.3 mm, 6.3 mm, 5.8 mm and 8.4 mm in class I, II, III and cleft groups respectively, being the distance in the latter significantly different. Angulation in the sagittal plane seems to play a role in terms of safety and properly developing the cut. Gulses *et al*. studied the angulation of the osteotome in the sagittal plane that caused less unfavorable fractures in 21 Turkish skulls, comparing angulations of +30°, 0° and −30° relative to the maxillary occlusal plane^42^. The +30° angulation (osteotome directed inferiorly) showed the highest number of high-level fractures. They concluded that positioning the osteotome parallel to the occlusal plane was the safest choice. Of note, Cheng and Robinson had previously recommended to always place the osteotome parallel to the maxillary occlusal plane to avoid injuries to the internal maxillary artery^28^. On the contrary, Girotto suggested that inferior angulation of the osteotome may minimize the propagation of fractures in a study about atypical fracture patterns of the pterygoid area^16^. Our study showed that, anatomically, the pterygomaxillary fissure has an angle greater than 90° to the maxillary occlusal plane (not perpendicular to it) for patients presenting class I, II or CLP, averaging 108.7°, 93.2° and 99.6° respectively. In class III patients, however, showed significantly smaller angles, averaging 84.4°. Whether placing the osteotome with the cutting end in this angulation decreases the occurrence of unfavorable fractures requires further clinical studies. Comparing the P and L levels, the L level is thicker but not wider. It is assumed that cutting and separating at the L level is more effective and safer. It is to be noted that the angle is larger in L level than that in P level. This information is helpful when we are inserting the osteotome and performing the PMJ disjunction.

The reported incidence of serious hemorrhage during LeFort I osteotomy is low. Kramer *et al*.^43^ prospectively studied 1000 patients, and described extensive bleeding requiring blood transfusion in 11 (1.1%) patients. Bleeding can frequently be due to penetrating trauma by an instrument or surgical handpiece, but also to posterior displacement of sharp bone fragments resulting from untoward fractures, injuring the soft tissue in the pterygopalatine fossa. Trauma to the pterygoid plexus is the most likely source of significant bleeding, whereas the maxillary artery and its terminal branches are most vulnerable to injure as they course through the pterygopalatine fossa. With regards to preventing hemorrhages, awareness of the safety distance from the osteotomy course to the major vessels is essential. It is not uncommon that during separation, the surgeon directs the osteotome too anteriorly, approaching the descending palatine artery. This artery can cause profuse bleeding, and most authors advise to preserve it whenever possible in order to decrease ischemic complications to the mobilized maxilla. We measured the distance from the pterygomaxillary junction to the greater palatine foramen, where the descending palatine artery passes through. The average of the distance was 2.8 ± 1.9 (class I), 3.2 ± 0.6 (class II), 3.0 ± 0.3 (class III) and 3.4 ± 1.0 mm (cleft group). Statistically significant difference was only found in the cleft group, who appears to have the greater palatine foramen more distant to the pterygomaxillary junction than the other groups. These distances are slightly shorter than those measured by Apinhasmit *et al*.^6^. This may be due to differences in the measurement method; we measured this distance at the axial cut at the level of the posterior nasal spine. On the other hand, damage to the pterygoid plexus can be minimized by avoiding the occurrence of pterygoid plate fractures, where bone fragments may be impacted affecting the surrounding soft tissue.

Despite the obvious evidence of increased risk in cleft patients following separation of pterygomaxillary junction, there has been only one study quantitatively supporting the anatomic differences of the cleft maxilla. Lee *et al*. studied the anatomy of the pterygomaxillary junction in cleft lip/palate patients, matching these patients with non-cleft Angle class III controls^44^. They concluded that cleft maxilla has greater width and thickness of the pterygomaxillary junction, and larger greater palatine foramen, with shorter medial pterygoid plate length than non-cleft class III patients. As mentioned previously, this greater pterygomaxillary thickness has been attributed to relatively high incidence of complications. This is supported by the study conducted by Wikkeling and Kopendraaier^20^, in which the ideal separations occurred in thin, edentulous bones. Our study, in contrast, shows that cleft patients present thinner junctions but higher incidence of unfavorable fractures at the posterior nasal spine level in the cleft side, particularly the fractures to the pterygoid plate. None of our patients, however, developed neurovascular complications related to the posterior maxilla at the time we reviewed the charts. This difference from the study by Lee *et al*. has yet to be explained; ethnicity or different surgical techniques for the primary repair of the cleft during childhood may be factors involved. Of note, the study by Lee *et al*. showed significant difference in both sides of the maxilla even in unilateral clefts compared to controls, while in the present study only the cleft side of the unilateral cleft was different.

Consistent with our results, several other researchers have found a negative correlation between the thickness of the pterygoid junction and the occurrence of the pterygoid plate fracture^5, 21, 22^. They hypothesized that when the pterygomaxillary junction is thin, the force from the osteotome may be easily dissipated leading to unwanted pterygoid plate fracture. One study suggested that pterygoid plate fractures in thin junctions did not advance upward to the skull base due to its low-level fracture nature^22^. It is possible that both extreme scenarios, particularly thick and particularly thin pterygomaxillary junctions, are at high risk. Thick junctions may be more prone to shatter erratically with an ascending and propagating course through the areas of low resistance, due to over excessive force exerted by the surgeon. It is still unknown the reason why cleft side in unilateral cleft lip/palate patients developed thinner junction, while bilateral cleft patients did not show this change. The abnormal maxillary morphology in the unilateral cleft patients has been suggested by Jiang *et al*.^45^, noticing that there was a significantly shortened maxillary length in the unilateral cleft compared with the normal control group at the dental level. It may be related to some asymmetric growth pattern that tends to collapse the cleft side, which does not happen in the bilateral cleft cases.

Syndromic patients with midface hypoplasia could receive a Le Fort III osteotomy and distraction in the mixed dentition age. The consequences of prior osteotomy through the PMJ as well as the formation of bony regenerate in this area can present significant challenge at later LeFort I osteotomy. Researchers have studied the pterygomaxillary region in both cadaveric samples^46^ and syndromic patients^47^ in order to better understand the anatomy in the area. As many of these patients will undergo a Le Fort 1 osteotomy once the growth is complete, surgeons should expect a thicker and irregular PMJ and a challenging surgical course in these patients.

We classified the pterygomaxillary separation in postoperative CBCT images at the posterior nasal spine level into three types: clean cut type, maxillary sinus type and the pterygoid plate fracture type. The results showed higher incidence of pterygoid plate fractures in the cleft patients, with the cleft side being the highest. Of note, none of our patients presented with neurovascular complications related to the posterior maxilla during the period comprised in the study. It is known that pterygoid plate fractures present with a higher incidence than other described complications^20, 40^. This risk is especifically associated to high-level untoward fractures approaching the skull base, whereas low-level fractures (below the horizontal osteotomy cut) seem to be of little clinical significance. The latter are undesired mainly because they tend to hamper the advancement of the maxilla due to the pterygoid muscle attachments to the plates. The injury mechanisms producing neurovascular complications can be direct, such as associated with bony impingement from an adjacent fracture site, but also by violation of the pterygopalatine fossa by sharp bone fragments, causing soft tissue damage. Perhaps future studies should focus on high level fractures. On the other hand, indirect injuries such as traction, compression, or contrecoup may be sustained during the process of disjunction itself^48^ which would explain why some cases of complications present with normal radiographic findings^17^. Up to this date, the relatively low number of reported cases of complications does not allow comprehensive study of the many factors involved.

With the introduction of virtual surgery planning, individual characteristics can be identified, and tailored treatment plans can be employed. However, not every center routinely uses CT imaging prior to the surgery, and an approximation of the population average anatomy would then be appreciated. An additional aim of this study was to evaluate the different anatomy in patients with cleft and malocclusion in general terms, which was accomplished.

A limitation of this study is that the force applied by the surgeon to the junction was not standardized or even measured. Therefore, it is impossible to evaluate to which extent this was a precipitating factor for occurrence of atypical fractures. Finally, the results in this study regarding angulation and positioning of osteotome were not necessarily followed during the surgeries, due to the retrospective nature of this study. Male gender and increased age has been reported to be a risk factor for the occurrence of pterygoid plate fractures^5–7^.

In summary, it can be concluded that there are no statistically significant differences in the thickness, width and angle of the PMJ relative to the sagittal plane between Angle’s classification I, II, III and cleft patients. The angle relative to the maxillary occlusal plane is smaller in non-cleft class III group compare to the other groups. Therefore, angulations and distances mentioned above are expected to be the safest when performing the pterygomaxillary disjunction. Differences displayed by cleft patients compared to the other groups are the significantly shorter distance to the distal aspect of the second molar, significantly longer distance between the posterior nasal spine and the lower border of the pterygomaxillary junction, and significantly thinner junction in the cleft side of the unilateral cleft group which may lead to higher incidence of pterygoid plate fracture. Moreover, the posterior maxilla in the cleft patients is shorter anteroposteriorly. This implies that, in these patients, the vertical cut should be made closer to the second molar. Also, cleft lip and palate patients show a slight, but significant, greater distance between the greater palatine foramen and the pterygomaxillary junction, thus preserving a slightly larger safety margin anteriorly.

Based on the results of this study, it can be concluded that surgeons should note that the cleft group may have different anatomical structures than normal patient group. Therefore, this study may help surgeons to prepare and plan the surgery using anatomical measurements as well as characteristics in the specific groups. Furthermore, it provides useful information in the selection of each group for performing a safer PMJ separation.

## Methods

### Study designs and patients

A retrospective study was done using the pre- and postoperative CBCT images of 283 consecutive patients who underwent orthognathic surgery at Chang Gung Craniofacial Center from January 2012 to December 2015. Patients were classified in groups according to their Angle classification (class I, class II, class III groups) and presence of cleft lip and palate (cleft group). This cleft group included patients with unilateral and bilateral cleft lip and palate. Both sides of the maxilla were studied. In the cleft group, each side was categorized as “cleft side” and “non-cleft side” for the cleft group. A total of 566 sides were evaluated. For each group, postoperative CBCT at average 1.5 months after the surgery were collected and axial images were evaluated at the level of the posterior nasal spine. Demographic data were described in Table 1. Exclusion criteria were previous facial trauma, surgery, tumor, or any other maxillofacial pathologic features other than the repaired cleft lip and palate in the cleft group. Patients without preoperative records or CBCT images were also excluded from the study. The study was approved by the Institutional Review Board of Chang Gung Medical Foundation (No. 201700088B0) and the study was performed following these guidelines. Informed consents were obtained from the patients or the parents.

### Surgical technique

The LeFort I osteotomy was performed by experienced surgeons in the center. For the pterygomaxillary separation, the pterygomaxillary groove was first identified with the tip of a curve osteotome, and then the osteotomy proceeded medially by tapping with a mallet. Digital pressure was used to identify possible remaining sites of resistance; in case these were present, further tapping was performed in the pterygomaxillary junction. After the disjunction was completed between the maxilla and the pterygoid process, maxillary downfracture was performed using Röwe disimpaction forceps.

### CBCT image analyses

The patient’s CBCT scanning was taken using an i-CAT^TM^ scanner (Imaging Sciences International, Hatfield, PA, USA) (voxel resolution: 0.4 mm) about 3 weeks prior to the scheduled surgery date. Data were stored in the Digital Image Communications in Medicine (DICOM) format. The DICOM data were analyzed with a commercial software program, Dolphin 3D (Dolphin Imaging & Management Solutions, Chatsworth, California, USA) reconstructed into multiple-plane views. Direct measurements were made with the computer software. In each patient, images from the identical axial level from each CBCT examination were selected by the examiner. Excel worksheets were designed to list the specific parameters. The data were then transferred to the computer for statistical analysis.

### Anatomical variables

**Figure 3:**
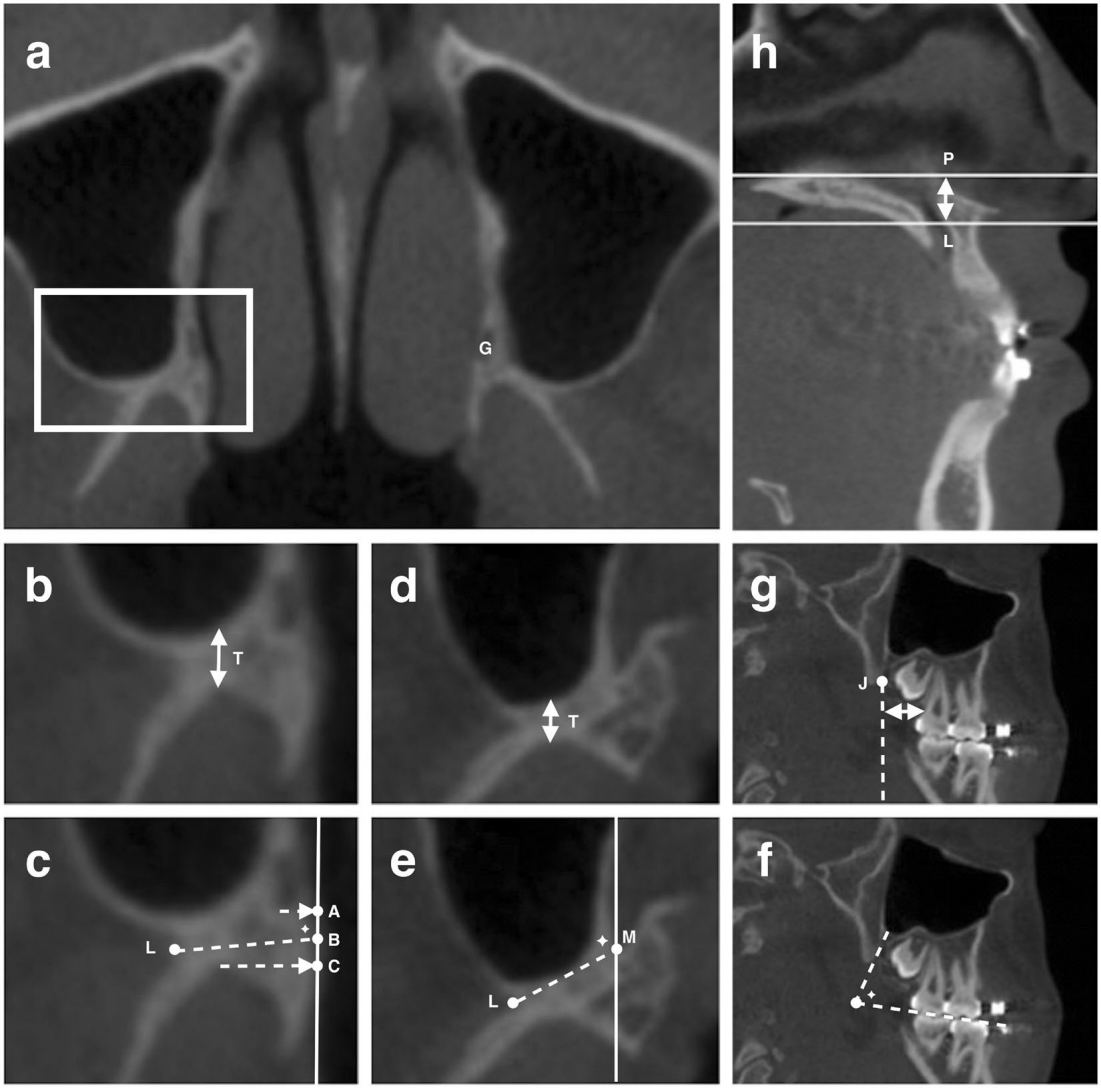
Landmarks for measurement in the pterygomaxillary junction (PMJ). (**a**) White square highlighting the PMJ region at the posterior nasal spine level (P level) in the axial view. The greater palatine foramen (G) is identified. (**b**) The thickness at the P level. The perpendicular distance from the most concave point of the pterygoid fossa to the posterior wall of the maxillary sinus. (**c**) At the P level, the width was between point L and point B; the angle relative to the sagittal plane (pointed star) was between the LB line and the sagittal line; and the distance between the greater palatine foramen and the PMJ was between the center of the greater palatine foramen and the LB line. (**d**) The thickness at the lower border of the PMJ level (L level): method same as (**b**). (**e**) At the L level, the width was between point L and point M; and the angle relative to the sagittal plane (pointed star) was between the LM line and the sagittal line. (**f**) The angle relative to the maxillary occlusal plane (pointed star) in the sagittal plane. (**g**) The distance between the perpendicular extension line created from the point J and the distal aspect of the root of the second molar. (**h**) The distance between the P level and the L level. Definition. Point A: The projection point from the lowest point of the greater palatine foramen to the medial surface of the PMJ. Point B: The midpoint between point A and C. Point C: The projection point from the most concave point of the pterygoid fossa to the sagittal line that passes through A. Point L: The most concave point of the lateral surface of PMJ. Point M: The most medial point of the PMJ in axial view. Point J: The lowest point of the PMJ in sagittal view.

In axial images, two levels were selected to measure the pterygomaxillary region, one at the posterior nasal spine level (P level) and another one at the lower border of PMJ (L level). The L level may not be exactly the same on both sides, depends on anatomic variation between sides. Measurements in the preoperative CBCT were described in Fig. 3.

### Pterygomaxillary separation type

**Figure 4:**
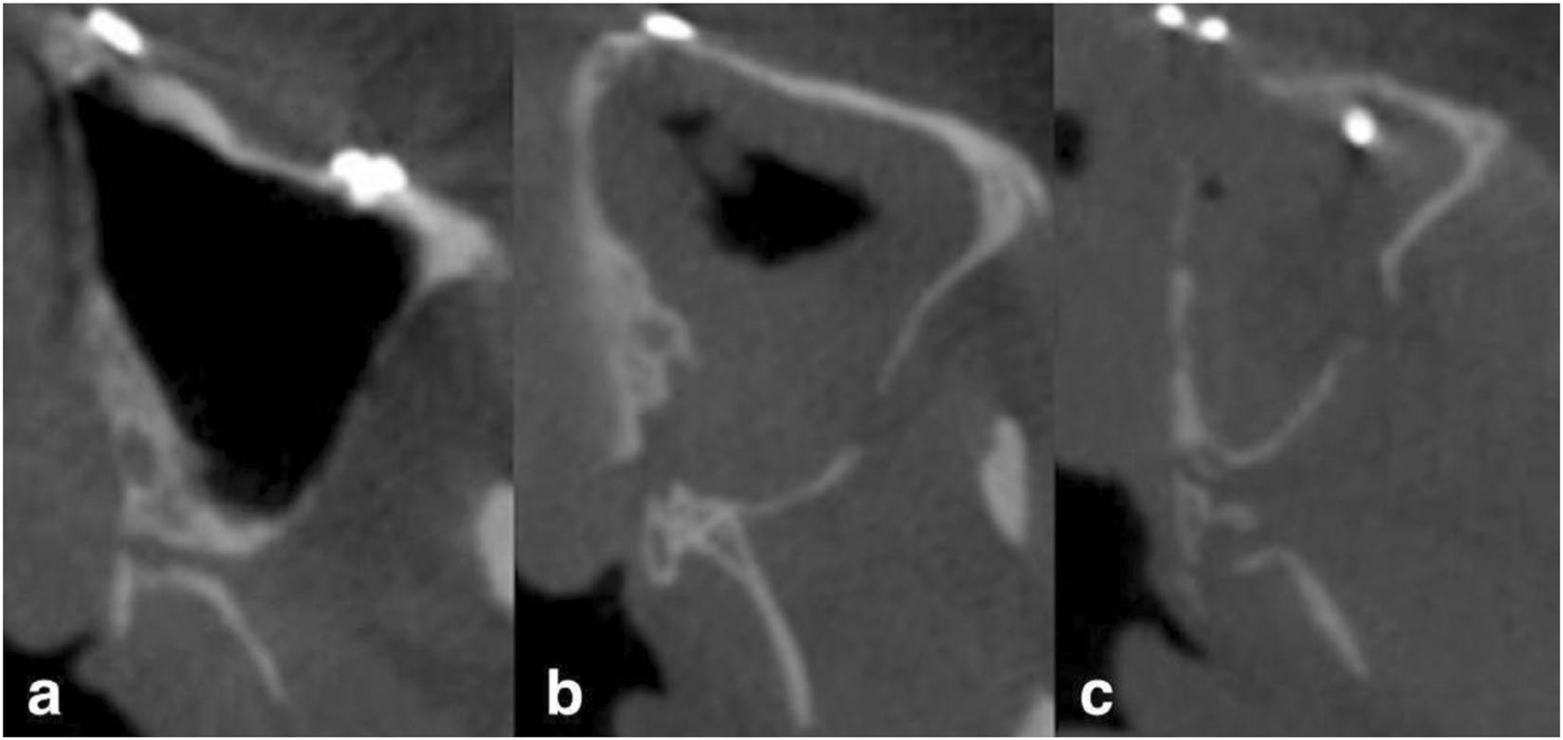
Pterygomaxillary separation type in postoperative CBCT. (**a**) Clean cut type: the cutting line within the PMJ. (**b**) Maxillary sinus type: part of the posterior wall of maxillary sinus attached to the PMJ after separation. (**c**) Pterygoid plate fracture type: pterygoid fracture occurred after PMJ separation.

Postoperative CBCT axial images were classified into three types defined as in Fig. 4: (a) Clean cut type: the cutting line right within the PMJ. This cutting type is most desirable for surgeons. (b) Maxillary sinus type: part of the posterior wall of the maxillary sinus was attached to the PMJ after separation. The osteotomy was too anterior. (c) Pterygoid plate fracture type: pterygoid fracture occurred after PMJ separation. The osteotomy was too posterior (Fig. 4).

### Error of the measurement

Reproducibility of the measurements was evaluated by comparing differences between the original and the repeated examinations of 10 randomly selected images in a one-week interval by the same examiner. The method error was defined as the reproducibility of double determination and it was calculated as the standard error of measurement. The method error of the repeated linear measurements was 0.3 mm (differences ranged from 0 to 0.4 mm). The method error in angular measurements was 3° (differences ranged from 1° to 5°). A statistically significant difference was not detected in neither linear nor angular measurements.

### Data analysis

All measurement data were tabulated and separated in the different groups. The mean, standard deviation (SD) and range for each measurement were calculated. Differences between groups were considered significant at p < 0.05. Data were analyzed and plotted with GraphPad Prism (version 5.00, La Jolla, USA). With the two levels of measurement defined as a within-group factor and different groups of malocclusion and cleft lip/palate group defined as a between-group factor, a two-way ANOVA test was performed to determine the effect of these factors on the anatomic variables measured in axial CT images. The distance to the second molar, the distance between the greater palatine foramen and the pterygomaxillary junction, and the distance between P level and L level were analyzed by one-way ANOVA. When a significant effect was observed, the Tukey test for one-way ANOVA and Bonferroni test for two-way ANOVA were used as a post hoc test to further characterize the significance of the specific differences. Statistical significance was set at p < 0.05. The chi-square test and Fisher exact test were performed to analyze the separation types of each group.

### Meeting presentation

The 11th Congress of the Asian Pacific Craniofacial Association, December 1-3, 2016, Nara, Japan.
